# Audio-Tokens: A toolbox for rating, sorting and comparing audio samples in the browser

**DOI:** 10.3758/s13428-022-01803-w

**Published:** 2022-03-16

**Authors:** Peter W. Donhauser, Denise Klein

**Affiliations:** 1 Cognitive Neuroscience Unit, Montreal Neurological Institute, McGill University, Montreal, QC H3A 2B4 Canada; 2 Ernst Strüngmann Institute for Neuroscience in Cooperation with Max Planck Society, 60528 Frankfurt am Main, Germany; 3 Centre for Research on Brain, Language and Music, McGill University, Montreal, QC H3G 2A8 Canada

**Keywords:** Online experiments, Auditory perception, Rating, JavaScript

## Abstract

Here we describe a JavaScript toolbox to perform online rating studies with auditory material. The main feature of the toolbox is that audio samples are associated with visual tokens on the screen that control audio playback and can be manipulated depending on the type of rating. This allows the collection of single- and multidimensional feature ratings, as well as categorical and similarity ratings. The toolbox (github.com/pwdonh/audio_tokens) can be used via a plugin for the widely used jsPsych, as well as using plain JavaScript for custom applications. We expect the toolbox to be useful in psychological research on speech and music perception, as well as for the curation and annotation of datasets in machine learning.

Collecting ratings from human participants is commonplace in behavioural research at multiple steps within a project. This includes validation and annotation of items to be included in a corpus (Ardila et al., 2020; Belin et al., 2008; Paquette et al., 2013) or stimulus material for neuroimaging experiments (Charest et al., 2014; Gold et al., 2019), but also to test hypotheses, such as perceptual differences between groups of participants (Atagi & Bent, 2016; Jack et al., 2012; Kutlu et al., 2021) or the effect of experimental conditions and stimulus properties (Holz et al., 2021; Lavan et al., 2016; McDermott et al., 2010; Thoret et al., 2021).

In research on speech and music, a common way to perform ratings is to first present an audio stimulus and subsequently present one or multiple rating scales (Belin et al., 2008; Holz et al., 2021). This sequential procedure requires the participant to hold the stimulus in working memory to perform the rating. This can be demanding for the participant, especially if multiple rating scales are used, or multiple audio stimuli are required to be rated at once. To alleviate this, a rating interface should allow the participant to control the playback of audio stimuli. At the same time, it is important that there is clear correspondence between playback controls and rating controls for individual audio stimuli, such as the multiple interfaces we outline below.

We present multiple rating interfaces in this paper that follow these constraints. They were inspired by two examples in the literature. The first one is called ‘auditory free classification’ (Clopper, 2008), which has been applied to non-native speech perception (Atagi & Bent, 2013, 2016). Participants are presented with a grid and multiple icons that control playback of audio stimuli from different speakers. These icons have to be arranged in the grid, forming as many groups of speakers as the participant thinks there are. The second example is based on a study on voice classification (Lavan et al., 2020), where participants were given a PowerPoint slide with embedded audio samples (represented as icons). They had to sort the icons on the slides into identities by clicking and dragging them. Instructions were either to sort them into two identities or choose the number of identities by themselves. Both these examples show creative ways of integrating audio playback controls in the rating/sorting interface. Here we expand on these ideas to generate rating interfaces that span a wide range of rating types. We implemented these in a toolbox called Audio-Tokens, which is easy to use and can be integrated within existing experimental workflows (both online and in-person studies).

## Description of the toolbox

**Fig. 1:**
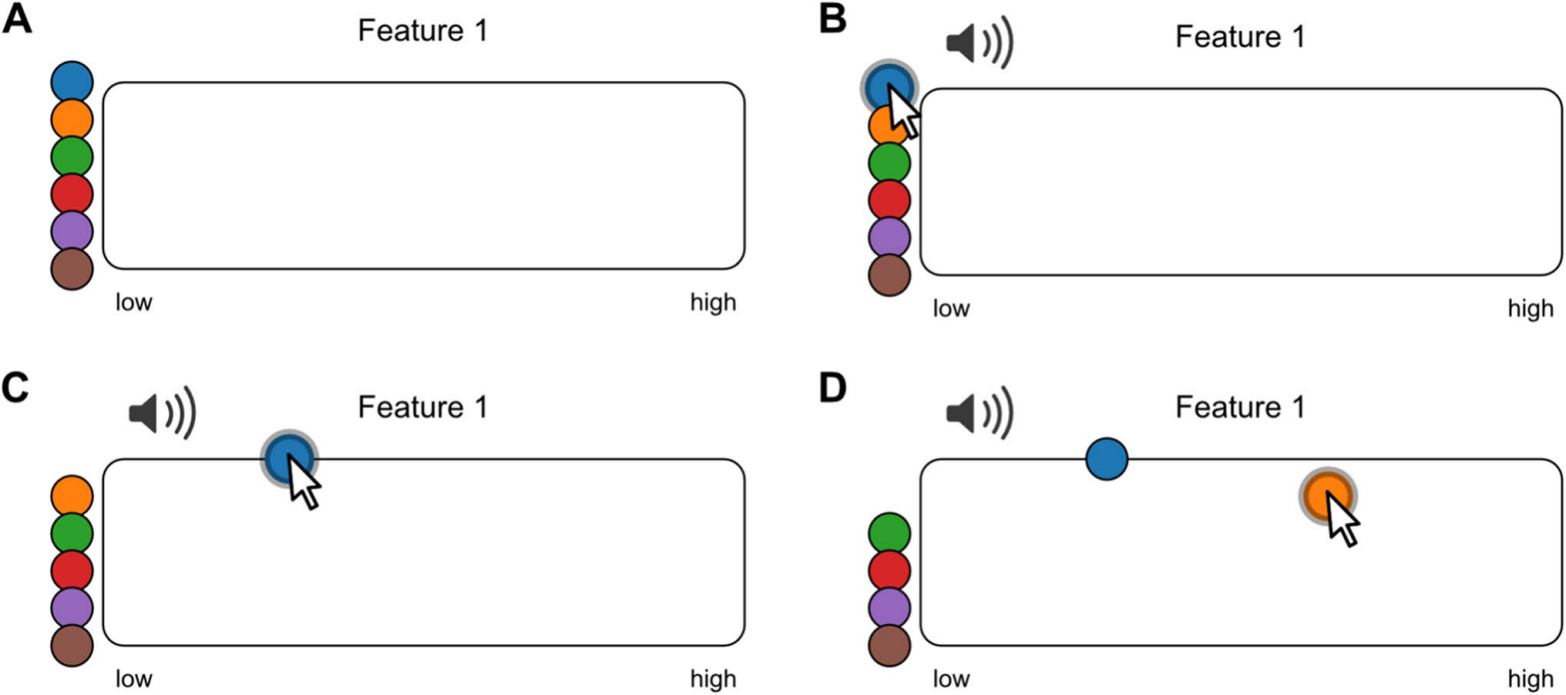
Rating audio samples along one feature. **a** Multiple coloured circles (*tokens*) are placed next to a rectangular *arena*. **b** Each token represents an audio stimulus which is played when hovering with the mouse. **c, d** Tokens can be dragged horizontally to place a rating. In the jsPsych plugin, use rating type: features. Use the following link for an interactive version: https://pwdonh.github.io/audio_tokens/index_query.html?type=single_feature

The interfaces we describe in this paper are based on the principle that each audio sample is represented by a coloured circle, which we will call a *token* (Fig. 1a). The user can control playback of the audio sample by hovering over the corresponding token (Fig. 1b). Ratings are performed by dragging tokens to different positions within an *arena* (here a rectangular box, Fig. 1c, d). In this paper, we introduce several rating types that are implemented in the toolbox by discussing some specific examples. The aim is to encourage researchers to use the tools in their own research.

### Continuous ratings

#### One feature

This first example (Fig. 1) shows the case of rating audio samples along one specified feature. This is likely one of the most common tasks a researcher would ask from participants. An example from speech research would be accent judgements. Participants are asked to listen to native or non-native speakers of a language and rate the strength of the foreign accent they perceive, either to study the process of second language learning with a focus on the speaker (Berken et al., 2016; Flege et al., 1995) or to examine social factors that determine what people consider ‘accented’ or standard variants of a language (Kutlu et al., 2021). The rating is implemented by dragging tokens within the *arena* horizontally (left-to-right position: low-to-high rating). When all tokens have been placed in the arena, participants are able to submit the ratings using a button.

A common problem in many studies that use sequential presentations of stimuli for rating is that participants might slowly drift in their ratings based on the stimuli presented earlier (Gerratt et al., 1993). The advantage of our approach is that it allows the simultaneous rating of multiple audio stimuli. As a consequence, participants can base their ratings on comparisons between the different stimuli. With the current method, a batch of speakers is presented at a time, thereby allowing the researcher to more tightly control the context of a given rating. For example, the same speaker can be presented in multiple randomly selected batches of speakers to average out the effects of context. In the current paper we are not trying to evaluate these effects of context, but we provide the tools to enable such studies.

The other rating interfaces in the toolbox follow the same principle. Different rating types are implemented by changing the arena layout and placement of tokens, constraining the dragging operations, as well as adding lines to represent connections between different tokens.

#### Multiple features

**Fig. 2:**
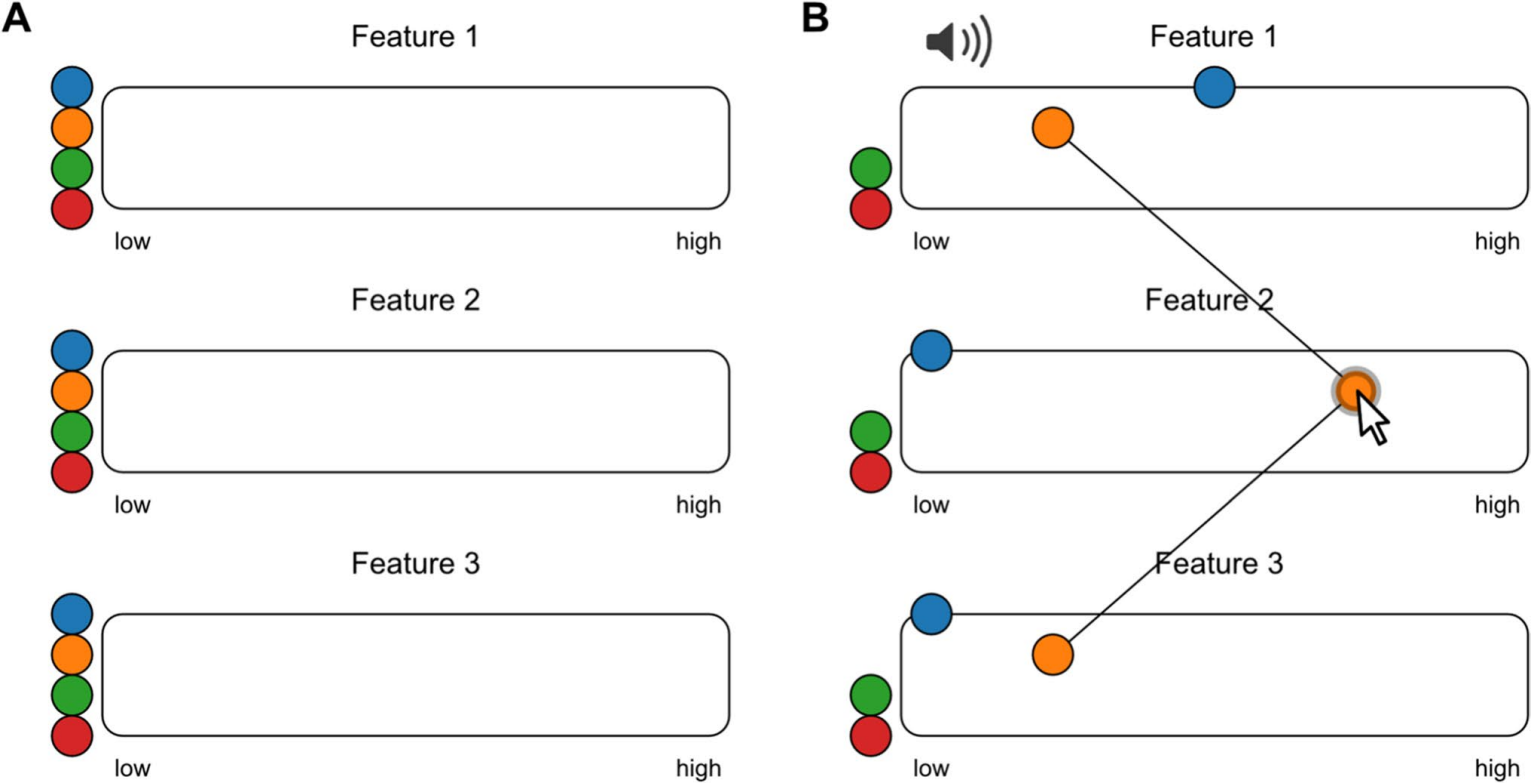
Rating audio samples along multiple features. **a** Three arenas are displayed corresponding to three different features to be rated. Tokens representing the same audio stimulus share colour and relative position. **b** Tokens can be individually dragged to place ratings for the different features. Lines will appear that connect corresponding tokens. In the jsPsych plugin, use rating type: features, while specifying multiple feature labels. Use the following link for an interactive version: https://pwdonh.github.io/audio_tokens/index_query.html?type=multiple_feature

The next interface allows for collecting ratings along multiple feature dimensions. An example of this would be rating clinically relevant features of speech impairments, such as voice quality, articulation and prosody (Darley et al., 1969; Mollaei et al., 2016). In the example shown in Fig. 2 there are three different *arenas*, which correspond to three feature dimensions. Tokens with the same colour represent the same audio file. Just like in the previous example, participants can drag tokens horizontally to place a rating. While hovering over a token, lines will appear that connect corresponding tokens (Fig. 2b). Again, in order to submit the ratings, all tokens have to be dragged into the arena.

Rating audio stimuli along multiple dimensions could be demanding for participants in a sequential set-up. In the interface offered here, participants can replay stimuli multiple times, and are helped by the matching colour and relative position of corresponding tokens. The connecting lines are an additional visual aid: instead of the average rating, they emphasise the profile across the rated features, e.g. the profile of a patients’ speech symptoms in the case of clinical speech evaluations. The connecting lines can be disabled, if that is more appropriate for the research question, e.g. if the participants should treat the features independently of each other.

**Fig. 3:**
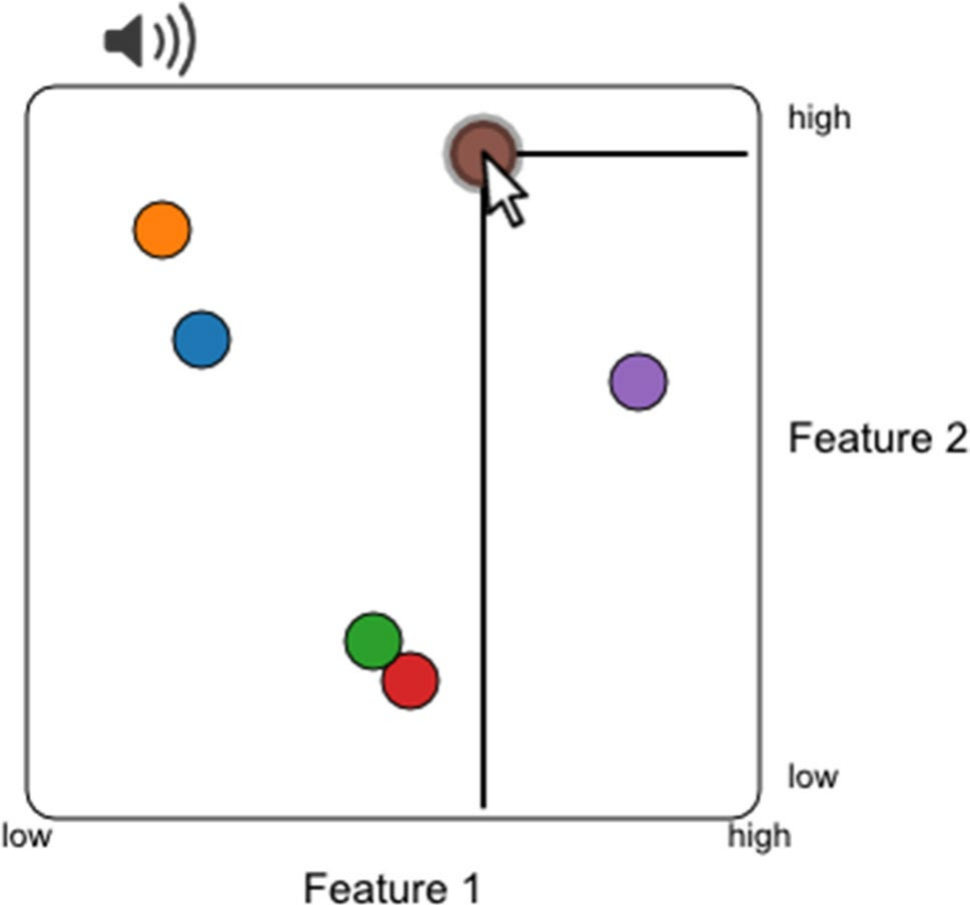
Rating audio samples along two features. Two-dimensional feature ratings can be placed in a 2D layout. In the jsPsych plugin, use rating type: features2d. Use the following link for an interactive version: https://pwdonh.github.io/audio_tokens/index_query.html?type=features2d

As a special case, the plugin allows ratings along two features to be performed in one arena (Fig. 3) by allowing both horizontal and vertical dragging operations. An example would be rating emotional content of speech or music along the dimensions valence and arousal (Holz et al., 2021; Paquette et al., 2013). Lines appear during dragging operations as a visual aid: they connect the current token to the corresponding positions on the vertical and horizontal axis.

### Categorical ratings

#### Fixed number of categories

**Fig. 4:**
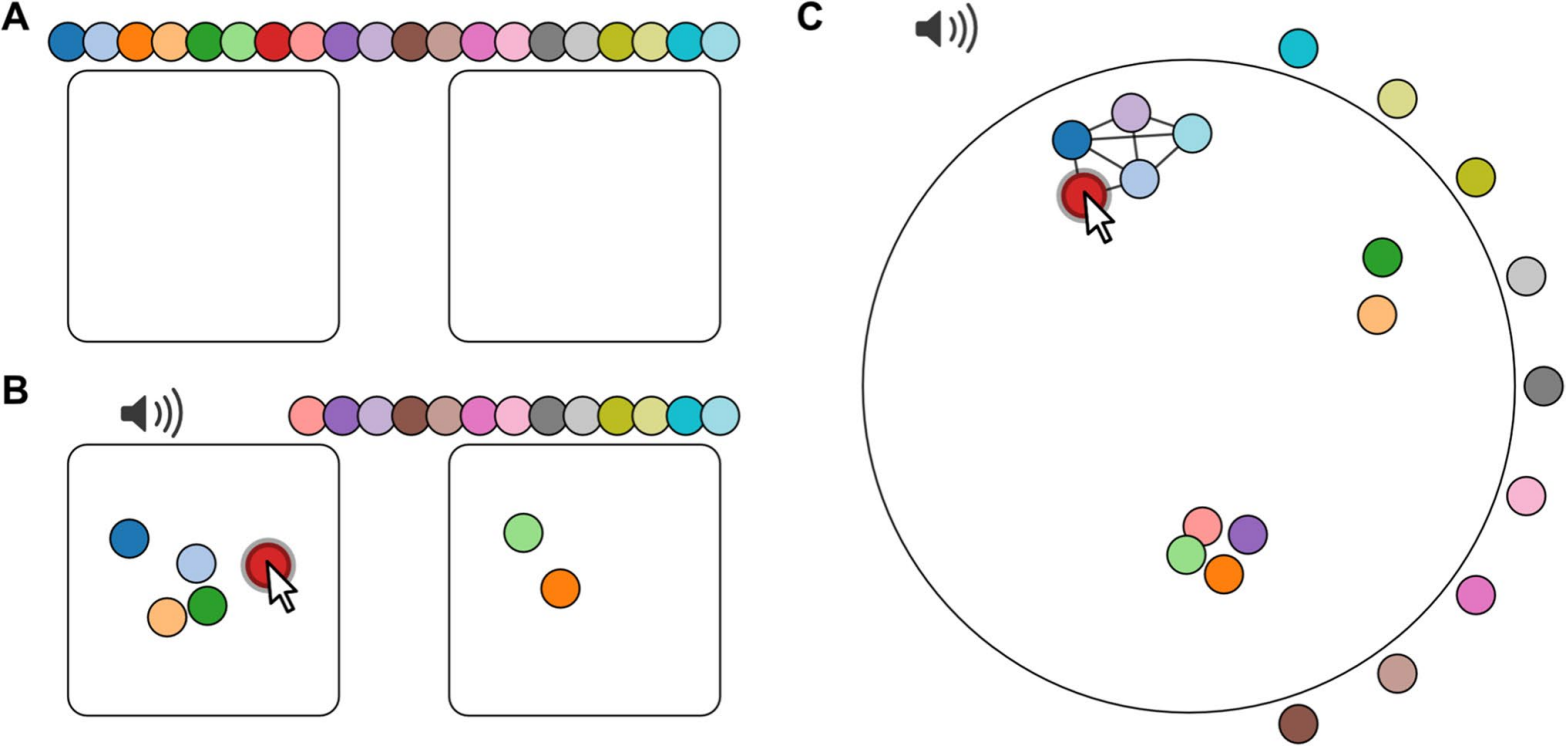
Sorting audio samples. **a** Fixed number of categories: Tokens are placed on top of two arenas representing two categories. **b** Tokens can be sorted by dragging them into one of the arenas. In the jsPsych plugin, use rating type: categories. **c** Variable number of categories: Tokens are placed around a circular arena and can be dragged freely inside it. Clusters are formed dynamically when tokens are dragged close to each other, highlighted by connecting lines. In the jsPsych plugin, use rating type: cluster. Use the following links for interactive versions: https://pwdonh.github.io/audio_tokens/index_query.html?type=categories, https://pwdonh.github.io/audio_tokens/index_query.html?type=cluster

Some research questions might require participants to sort audio recordings into discrete categories. In one scenario, the categories are specified by the experimenter, for example in quality control (keep or reject) or voice identity sorting (Lavan et al., 2020): participants are asked to sort utterances recorded from two different speakers. In this interface, several tokens will appear above two arenas (here labelled as ‘Category 1’ and ‘Category 2’, Fig. 4a, b). Tokens can be placed in the two arenas representing the two categories. The interface does not record where in the arena the tokens are dropped, because this is a discrete rating. Categories can be labelled as shown below, or they can be left blank if the question is less constrained (‘Please sort these recordings into two piles’).

#### Variable number of categories

In another scenario, both the number of categories and their labels are kept unspecified. Participants are asked to sort the recordings into as many categories as deemed appropriate. For example, Bent and colleagues (Bent et al., 2016) asked participants to group speakers of different regional or non-native accents by perceived origin.

In this interface (Fig. 4c), tokens are placed around a circular arena, and can be dragged freely within it. Clusters are formed dynamically when tokens are placed close to each other: all tokens of the current cluster are highlighted by connecting lines. Again, the interface will record only the category (cluster) membership and not the exact placement of the tokens, because this is a discrete rating.

### Similarity ratings

#### Unconstrained similarity rating

**Fig. 5:**
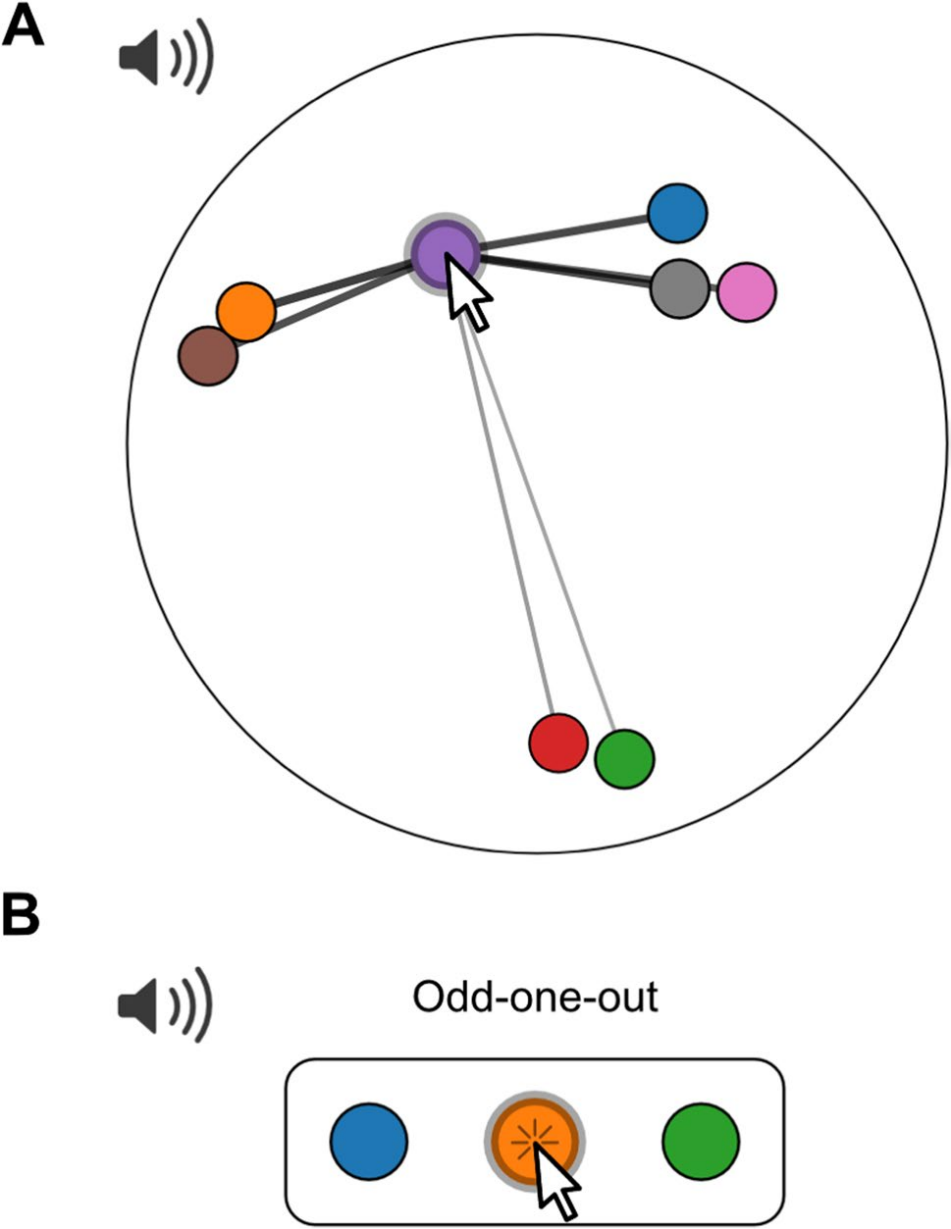
Rating the similarity of audio samples. **a** Unconstrained similarity rating: The interface looks similar to Fig. 4c; however, the actual position of tokens is recorded rather than category membership. Connecting lines emphasize the distance between tokens via the stroke width. In the jsPsych plugin, use rating type: similarity. **b** Relative similarity rating: Three tokens are displayed at a time. Participants are asked to select the audio stimulus that is least similar to the other two by clicking on the corresponding token. In the jsPsych plugin, use rating type: triplets. Use the following links for interactive versions: https://pwdonh.github.io/audio_tokens/index_query.html?type=similarity, https://pwdonh.github.io/audio_tokens/index_query.html?type=triplets

As the most unconstrained form of rating, we provide an interface for similarity ratings where participants are free to place tokens in a circular arena (Fig. 5a), similar to what has been used in the visual domain (Charest et al., 2014; Kriegeskorte & Mur, 2012). In contrast to the interface described above, the actual placement coordinates are recorded in the results. In addition, connecting lines are drawn between the currently dragged token and all others, while stroke width is scaled by the distance to the other tokens: this serves as a visual analogue for stimulus similarity.

#### Relative similarity rating in the triplet task

The last option is to ask participants for judgements on relative similarity (Anikin et al., 2018; Hebart et al., 2020; Raijmakers et al., 2004): Out of three stimuli, which one is the least similar to the other two (the odd one out). This interface differs from the others, since there are no dragging operations. Instead, three tokens are shown on the screen at a given time, and the odd one out is selected by a button click. If a trial consists of only three stimuli, it ends here. If there are more than three stimuli, the selected token will disappear and a new token will appear in its place. The participant can make a new decision: which one is now the odd one out. Since the decisions are made by mouse click, the trial ends automatically and there is no submit button.

A similarity rating as in Fig. 5a can be demanding for the participant, especially as the number of tokens increases. Stimuli might differ on many dimensions, but the two-dimensional layout will require participants to ignore some of those dimensions. For placement of a token, participants have to simultaneously consider the similarity of the current to all the other stimuli, which could be highly demanding. The second task (Fig. 5b) is deliberately much simpler: the participant has to compare only three different stimuli and make a discrete judgement. This is a less expressive type of rating, but might be much easier and faster for participants to perform.

## Usage of the toolbox

### Implementation options

The basic tools are written in JavaScript, because it is the predominant scripting language for the web. The graphical elements are drawn in the browser as SVG using the data visualization library D3^1^. The rating tools can thus be flexibly used in any type of environment (e.g. personal or institutional web page). We tested functionality for Chrome and Firefox browsers.

More convenient than using plain JavaScript, we implemented the toolbox as a plugin within the widely used jsPsych framework for running online experiments (de Leeuw, 2015). This means our tools can be easily integrated into existing behavioural experiments running on jsPsych. There are also multiple options of hosting jsPsych experiments such as Pavlovia^2^, Jatos^3^ and others, all of which are described in the official jsPsych documentation^4^. Although built for online experiments, all experiments written in jsPsych can be performed in a laboratory setting as well, using a browser on a local computer.

### Example script for jsPsych plugin

We go through the basic steps of setting up an experiment here. The complete experiment can be written in one HTML file. This is the minimal page set-up, excluding the jsPsych code:


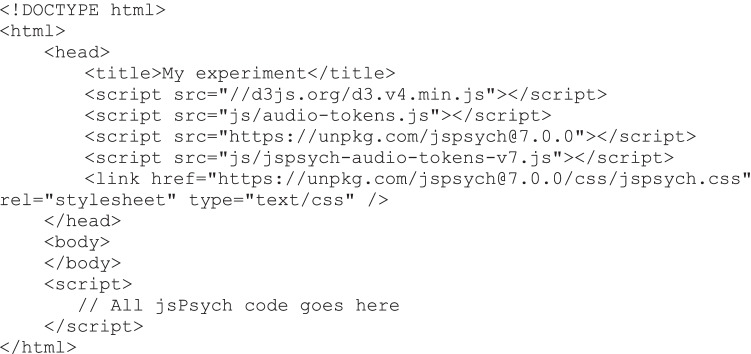


Here we are first importing a few libraries:

- d3.v4.min.js: This is the D3 library on which the current toolbox is based; it is widely used for visualizing data in web environments.
- audio-tokens.js: This is the JavaScript code for the rating tools described in this paper.
- jspsych.js: This provides all the basic functionality of jsPsych.
- jspsych-audio-tokens-v7.js: This is the plugin that allows us to include the rating tools in a jsPsych experiment.
- More jsPsych plugins can be loaded here in order to build your experiment, e.g. to display instructions.

There is nothing written in between the <body></body> tags. This is normally where the content of a web page goes: here jsPsych takes care of displaying content according to the trial structure of the experiment.

In between the <script></script> tags we will add all the jsPsych code:

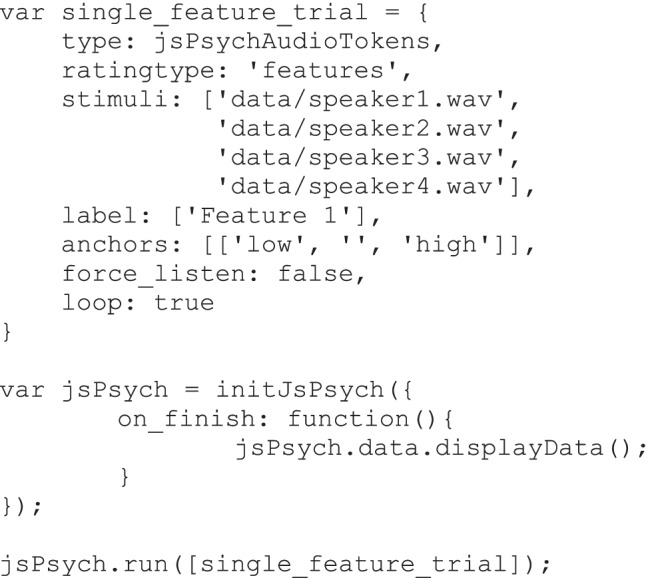



Here, single_feature_trial is the variable holding the parameters for a jsPsych trial:

- type: Here we tell jsPsych to display a trial using our plugin called jsPsychAudioTokens.
- ratingtype: This tells our plugin what rating type to use among the ones described in this paper. Options are: features, features2d, categories, cluster, similarity, triplets.
- stimuli: This is an array containing the file paths of the audio stimuli for this trial relative to the directory where the HTML file is stored.
- label: This specifies the label to be displayed for a given rating dimension (e.g. valence, arousal, accentedness). See Fig. 1, the label on top of the arena.
- anchors: This specifies the labels displayed as the endpoints of the rating dimensions (e.g. low-high, positive-neutral-negative).
- force_listen: This, if set to true, checks whether the participant has listened to the whole audio file before allowing them to submit their ratings.
- loop: If set to true, the audio file will be played in a loop while hovering over a token.

The call to initJsPsych initializes jsPsych and jsPsych.run starts the experiment. In this case, the experiment timeline includes only one trial and the recorded data will be presented on the screen after the experiment is finished. The data is by default formatted in JSON, and for the example trial looks like the following:

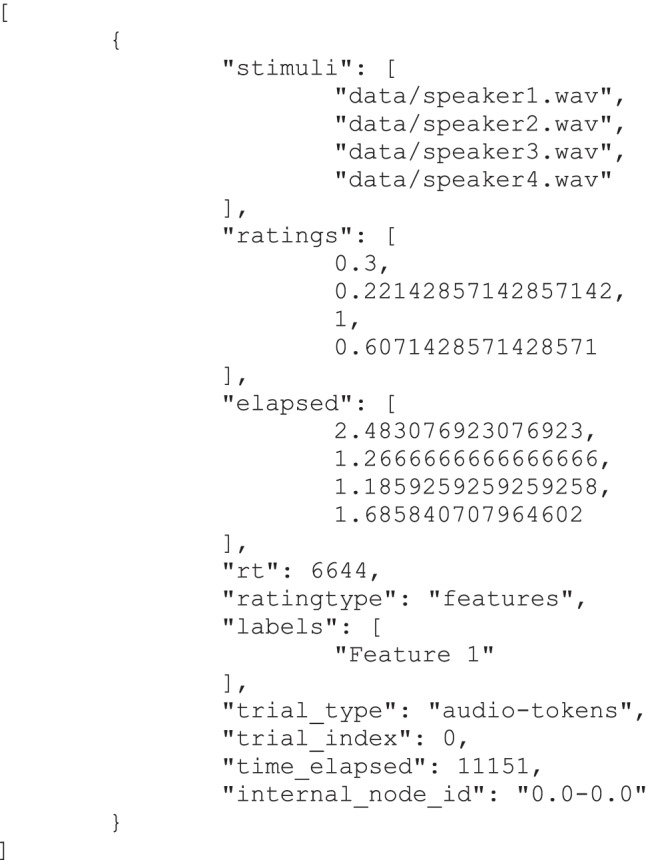



The following fields are specific to our plugin:

- ratings: An array containing the rating the participant gave. This differs depending on the rating type. Here we get a number between 0 and 1 representing the horizontal placement of each token (0: left, 1: right).
- elapsed: An array containing the number of times a stimulus has been played by the participant. In this example, the first stimulus was played approximately two and a half times. If the audio playback is set to loop mode (see above), this can include the time that it took the participant to place the rating, rather than intentional playback.
- rt: The time, in milliseconds, from starting the trial to submitting the ratings.

The fields stimuli, ratingtype, labels and anchors are equivalent to the parameters specified in the experiment script. The remaining fields are generic jsPsych outputs.

Please refer to our GitHub repository^5^ for additional examples and the jsPsych documentation on multiple ways to save the data. In the GitHub repository, we provide a Python command-line tool to convert the results coming from jsPsych into a spreadsheet that is more convenient to use for further statistical analysis.

## Conclusions

In this paper, we presented a new toolbox for collecting human ratings of audio samples through the browser. We think it is unique in the range of options that are available: using a consistent interface design, participants can be asked to rate audio stimuli on one or multiple feature dimensions, sort audio stimuli into two or more categories, or compare sets of audio stimuli using e.g. an odd-one-out procedure. As such, it adds substantial flexibility in the design of rating studies, which would otherwise be constrained by the sequential nature of the task (first listen then rate). In addition, it is possible to extend the toolbox to novel applications while preserving the basic principle: for example, by changing arena layout, changing token placement, setting different constraints on dragging operations or allowing continuous manipulation of stimulus features through dragging operations (see e.g. Harrison et al., 2020). In conclusion, we hope that researchers will take advantage of the flexibility offered by the toolbox and that it can help generate new experimental designs and research questions.

## Footnotes

1 d3js.org

2 pavlovia.org

3 www.jatos.org

4 www.jspsych.org

5 github.com/pwdonh/audio_tokens

## References

[CR1] Anikin A, Bååth R, Persson T (2018). Human Non-linguistic Vocal Repertoire: Call Types and Their Meaning. Journal of Nonverbal Behavior.

[CR2] Ardila, R., Branson, M., Davis, K., Kohler, M., Meyer, J., Henretty, M., … Weber, G. (2020). Common voice: a massively-multilingual speech corpus. *Proceedings of the 12th Language Resources and Evaluation Conference*, 4218–4222.

[CR3] Atagi, E., & Bent, T. (2013). Auditory free classification of nonnative speech. Journal of Phonetics, 41(6), 509–519. 10.1016/j.wocn.2013.09.003 PMC3867985 24363470

[CR4] Atagi E, Bent T (2016). Auditory free classification of native and nonnative speech by nonnative listeners. Applied Psycholinguistics.

[CR5] Belin P, Fillion-Bilodeau S, Gosselin F (2008). The Montreal Affective Voices: a validated set of nonverbal affect bursts for research on auditory affective processing. Behavior Research Methods.

[CR6] Bent T, Atagi E, Akbik A, Bonifield E (2016). Classification of regional dialects, international dialects, and nonnative accents. Journal of Phonetics.

[CR7] Berken JA, Gracco VL, Chen J-K, Klein D (2016). The timing of language learning shapes brain structure associated with articulation. Brain Structure & Function.

[CR8] Charest I, Kievit RA, Schmitz TW, Deca D, Kriegeskorte N (2014). Unique semantic space in the brain of each beholder predicts perceived similarity. Proceedings of the National Academy of Sciences of the United States of America.

[CR9] Clopper CG (2008). Auditory free classification: methods and analysis. Behavior Research Methods.

[CR10] Darley FL, Aronson AE, Brown JR (1969). Differential diagnostic patterns of dysarthria. Journal of Speech and Hearing Research.

[CR11] de Leeuw JR (2015). jsPsych: a JavaScript library for creating behavioral experiments in a Web browser. Behavior Research Methods.

[CR12] Flege JE, Munro MJ, MacKay IR (1995). Factors affecting strength of perceived foreign accent in a second language. The Journal of the Acoustical Society of America.

[CR13] Gerratt BR, Kreiman J, Antonanzas-Barroso N, Berke GS (1993). Comparing internal and external standards in voice quality judgments. Journal of Speech and Hearing Research.

[CR14] Gold BP, Mas-Herrero E, Zeighami Y, Benovoy M, Dagher A, Zatorre RJ (2019). Musical reward prediction errors engage the nucleus accumbens and motivate learning. Proceedings of the National Academy of Sciences of the United States of America.

[CR15] Harrison, P., Marjieh, R., Adolfi, F., van Rijn, P., Anglada-Tort, M., Tchernichovski, O., ... & Jacoby, N. (2020). Gibbs sampling with people. *Advances in Neural Information Processing Systems*, 33, 10659–10671.

[CR16] Hebart MN, Zheng CY, Pereira F, Baker CI (2020). Revealing the multidimensional mental representations of natural objects underlying human similarity judgements. Nature Human Behaviour.

[CR17] Holz N, Larrouy-Maestri P, Poeppel D (2021). The paradoxical role of emotional intensity in the perception of vocal affect. Scientific Reports.

[CR18] Jack RE, Garrod OGB, Yu H, Caldara R, Schyns PG (2012). Facial expressions of emotion are not culturally universal. Proceedings of the National Academy of Sciences of the United States of America.

[CR19] Kriegeskorte N, Mur M (2012). Inverse MDS: Inferring Dissimilarity Structure from Multiple Item Arrangements. Frontiers in Psychology.

[CR20] Kutlu, E., Tiv, M., Wulff, S., & Titone, D. (2021). The impact of race on speech perception and accentedness judgements in racially diverse and non-diverse groups. *Applied Linguistics*, amab072.

[CR21] Lavan N, Scott SK, McGettigan C (2016). Laugh Like You Mean It: Authenticity Modulates Acoustic, Physiological and Perceptual Properties of Laughter. Journal of Nonverbal Behavior.

[CR22] Lavan N, Merriman SE, Ladwa P, Burston LFK, Knight S, McGettigan C (2020). “Please sort these voice recordings into 2 identities”: Effects of task instructions on performance in voice sorting studies. British Journal of Psychology.

[CR23] McDermott JH, Lehr AJ, Oxenham AJ (2010). Individual differences reveal the basis of consonance. Current Biology: CB.

[CR24] Mollaei F, Shiller DM, Baum SR, Gracco VL (2016). Sensorimotor control of vocal pitch and formant frequencies in Parkinson’s disease. Brain Research.

[CR25] Paquette S, Peretz I, Belin P (2013). The “Musical Emotional Bursts”: a validated set of musical affect bursts to investigate auditory affective processing. Frontiers in Psychology.

[CR26] Raijmakers MEJ, Jansen BRJ, van der Maas HLJ (2004). Rules and development in triad classification task performance. Developmental Review.

[CR27] Thoret, E., Caramiaux, B., Depalle, P., & Mcadams, S. (2021). Learning metrics on spectrotemporal modulations reveals the perception of musical instrument timbre. *Nature Human Behaviour*, 5(3), 369–377. 10.1038/s41562-020-00987-5 33257878

